# Methionine metabolism and methyltransferases in the regulation of aging and lifespan extension across species

**DOI:** 10.1111/acel.13034

**Published:** 2019-08-28

**Authors:** Andrey A. Parkhitko, Patrick Jouandin, Stephanie E. Mohr, Norbert Perrimon

**Affiliations:** ^1^ Department of Genetics Blavatnik Institute Harvard Medical School Boston Massachusetts; ^2^ Howard Hughes Medical Institute Boston Massachusetts

**Keywords:** aging, lifespan, methionine restriction, methylation, methyltransferases, *S*‐adenosylmethionine

## Abstract

Methionine restriction (MetR) extends lifespan across different species and exerts beneficial effects on metabolic health and inflammatory responses. In contrast, certain cancer cells exhibit methionine auxotrophy that can be exploited for therapeutic treatment, as decreasing dietary methionine selectively suppresses tumor growth. Thus, MetR represents an intervention that can extend lifespan with a complementary effect of delaying tumor growth. Beyond its function in protein synthesis, methionine feeds into complex metabolic pathways including the methionine cycle, the transsulfuration pathway, and polyamine biosynthesis. Manipulation of each of these branches extends lifespan; however, the interplay between MetR and these branches during regulation of lifespan is not well understood. In addition, a potential mechanism linking the activity of methionine metabolism and lifespan is regulation of production of the methyl donor *S*‐adenosylmethionine, which, after transferring its methyl group, is converted to *S*‐adenosylhomocysteine. Methylation regulates a wide range of processes, including those thought to be responsible for lifespan extension by MetR. Although the exact mechanisms of lifespan extension by MetR or methionine metabolism reprogramming are unknown, it may act via reducing the rate of translation, modifying gene expression, inducing a hormetic response, modulating autophagy, or inducing mitochondrial function, antioxidant defense, or other metabolic processes. Here, we review the mechanisms of lifespan extension by MetR and different branches of methionine metabolism in different species and the potential for exploiting the regulation of methyltransferases to delay aging.

## 1 METHIONINE METABOLISM

**Figure 1:**
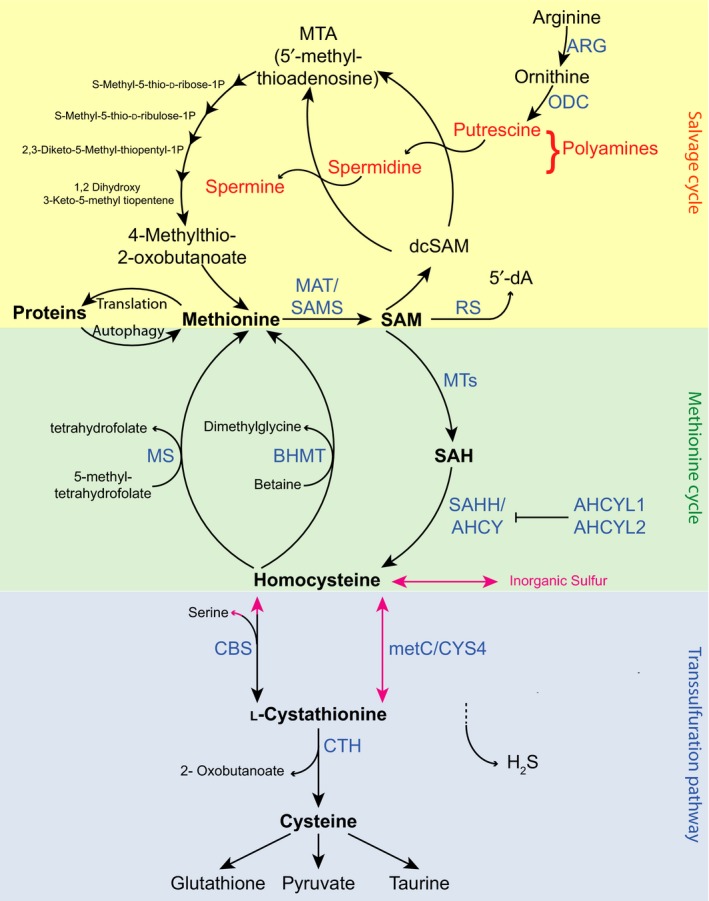
Schematic of methionine metabolism

Methionine is an essential proteogenic amino acid necessary for normal growth and development and is one of the four common sulfur‐containing amino acids (methionine, cysteine, homocysteine, and taurine). In humans, methionine can be obtained from food or from gastrointestinal microbes. Methionine plays a well‐known role as an initiator of protein synthesis in prokaryotes and eukaryotes and functions as an endogenous antioxidant at the surface of proteins (Levine, Mosoni, Berlett, & Stadtman, 1996; Luo & Levine, 2009). In addition, methionine serves major roles through its metabolism, which fuels a variety of metabolic pathways. Methionine metabolism can be broken into three parts: the methionine cycle, the transsulfuration pathway, and the salvage cycle (Figure 1).

### 1.1 Methionine cycle

The first step in methionine metabolism is performed by methionine adenosyltransferase (MAT), an enzyme conserved from *Escherichia coli* to humans that catalyzes the biosynthesis of *S*‐adenosylmethionine (SAM) from methionine and ATP. SAM is the principal methyl donor and the second most widely used enzyme substrate after ATP (Cantoni, 1975). During substrate methylation, SAM donates its methyl group to acceptor molecules, for example, DNA, RNA, proteins, or other cellular metabolites, generating *S*‐denosylhomocysteinee (SAH). Over 200 known or putative methyltransferases have been identified in the human genome (Petrossian & Clarke, 2011) and 81 in yeast (Petrossian & Clarke, 2009). *S*‐denosylhomocysteine hydrolase (SAHH/AHCY) catalyzes the reversible hydrolysis of SAH to adenosine and l‐homocysteine. SAHH/AHCY proteins are tetramers with a NADH/NAD^+^ cofactor bound in the active site of each subunit (Brzezinski, Bujacz, & Jaskolski, 2008). There are also two AHCY‐like proteins, AHCYL1 and AHCYL2, which most likely have lost their canonical enzymatic functions due to critical mutations in their AHCY domains. However, via hetero‐multimerization, ACHYL1 and AHCYL2 can suppress the enzymatic activity of AHCY and thus act as dominant negative regulators of canonical AHCY (Devogelaere, Sammels, & Smedt, 2008). Cells must maintain low concentrations of SAH, which is a product inhibitor of SAM‐dependent methylation reactions. Methyltransferases catalyze a variety of methylation reactions via the transfer of methyl groups on histone proteins as well as to nucleic acids, nonhistone proteins, and metabolites, although different methyltransferases exhibit different sensitivity to inhibition by SAH (Huang et al., 2000). Homocysteine can be remethylated to form methionine and retained in the methylation cycle, or converted to cysteine via the transsulfuration pathway and thus withdrawn from the methylation cycle. Remethylation of homocysteine to form methionine completes the methionine cycle. This process involves either methionine synthase (MS), which requires 5‐methyltetrahydrofolate as a methyl donor, or betaine homocysteine methyltransferase (BHMT), which requires betaine as a methyl donor.

### 1.2 Transsulfuration pathway

Homocysteine from the methionine cycle can also be utilized in the transsulfuration pathway to produce cysteine. Cystathionine‐β‐synthase is the first and rate‐limiting enzyme of the transsulfuration pathway, the primary metabolic pathway for the synthesis of cysteine. Cystathionine‐β‐synthase synthesizes cystathionine from the condensation of homocysteine and serine. Cystathionine is hydrolyzed by cystathionine‐γ‐lyase to produce cysteine, which is further used in the synthesis of proteins, glutathione, and taurine. Cystathionine‐γ‐lyase and cystathionine‐β‐synthase also catalyze the production of hydrogen sulfide (H_2_S) from cysteine and homocysteine. H_2_S is a signaling molecule and cytoprotectant with a wide range of physiological functions. H_2_S protects cells from oxidative stress and can modulate neuronal transmission, smooth muscle relaxation, release of insulin, and the inflammatory response. Activation of the transsulfuration pathway promotes the production of H_2_S (Kabil, Vitvitsky, & Banerjee, 2014; Wallace & Wang, 2015).

### 1.3 Salvage pathway and polyamine biosynthesis

The methionine salvage pathway, or 5′‐methylthioadenosine (MTA) cycle, regenerates methionine from SAM and is responsible for the production of polyamines (Minois, Carmona‐Gutierrez, & Madeo, 2011; Pegg, 2016). In the methionine salvage pathway, SAM is decarboxylated by AdoMet decarboxylase into decarboxylated SAM (dcSAM), which serves as an aminopropyl group donor. In parallel, arginase converts arginine into ornithine, which is then decarboxylated by ornithine decarboxylase (ODC) to produce putrescine. Putrescine is further converted to spermidine and spermine through the consecutive action of two distinct aminopropyl transferases, spermidine synthase and spermine synthase, which use dcSAM as an aminopropyl donor. dcSAM is converted to MTA after the donation of an aminopropyl group for polyamine synthesis, and MTA is converted via six enzymatic steps back to methionine (Minois et al., 2011; Pegg, 2016). In addition to participating in methylation and synthesis of polyamines, SAM can also be activated by members of the radical SAM superfamily of enzymes that convert SAM to a highly oxidizing 5′‐deoxyadenosyl radical intermediate involved in a variety of reactions (Landgraf, McCarthy, & Booker, 2016).

Beside its proteogenic and metabolic roles, methionine can also serve as an antioxidant. Methionine is one of the major targets of reactive oxygen species (ROS). Surface‐exposed methionine residues of native proteins can be oxidized by ROS to R‐ and *S*‐methionine sulfoxide, which can be reduced back to methionine by methionine sulfoxide reductases.

Consistent with the importance of methionine metabolism in cellular physiology, dysregulation of methionine metabolism has been reported in multiple diseases. Moreover, methionine restriction (MetR) has been tested as a treatment for disease in a number of clinical trials. Six genetic conditions in humans are known to lead to methionine and homocysteine elevation, that is, hypermethioninemias and hyperhomocysteinemias, as a result of deficiencies of enzymes involved in methionine metabolism (MAT, CBS, GNMT, AHCY) or affecting the mitochondrial transporter citrin and fumarylacetoacetate hydrolase (FAH; Mudd, 2011). Hyperhomocysteinemia is a characteristic of aging and upregulated levels of homocysteine contribute to the pathogenesis of multiple age‐associated diseases, including cardiovascular dysfunction, decline in renal and cognitive functions, bone fractures, and others (Ostrakhovitch & Tabibzadeh, 2019). However, under certain circumstances hyperhomocysteinemia can be observed without any pathological changes; this raises a question whether homocysteine contributes to pathogenesis or only serves as a biomarker. In addition, multiple cancer types are auxotrophic for methionine due to changes in methionine metabolism (Agrawal, Alpini, Stone, Frenkel, & Frankel, 2012; Cavuoto & Fenech, 2012), a feature further exploited in a variety of therapeutic approaches (clinicaltrials.gov). In addition, ^11^C‐methionine is the most popular amino acid tracer for PET imaging of brain tumors (Glaudemans et al., 2013). Finally, MetR in rodents not only extends lifespan but also protects from visceral fat mass accumulation and from the negative effects of a high‐fat diet (Ables, Perrone, Orentreich, & Orentreich, 2012; Malloy et al., 2006; Orentreich, Matias, DeFelice, & Zimmerman, 1993; Wanders et al., 2018). Based on these results, MetR has also been tested in clinical trials of obese adults with metabolic syndrome (Plaisance et al., 2011).

**Figure 2:**
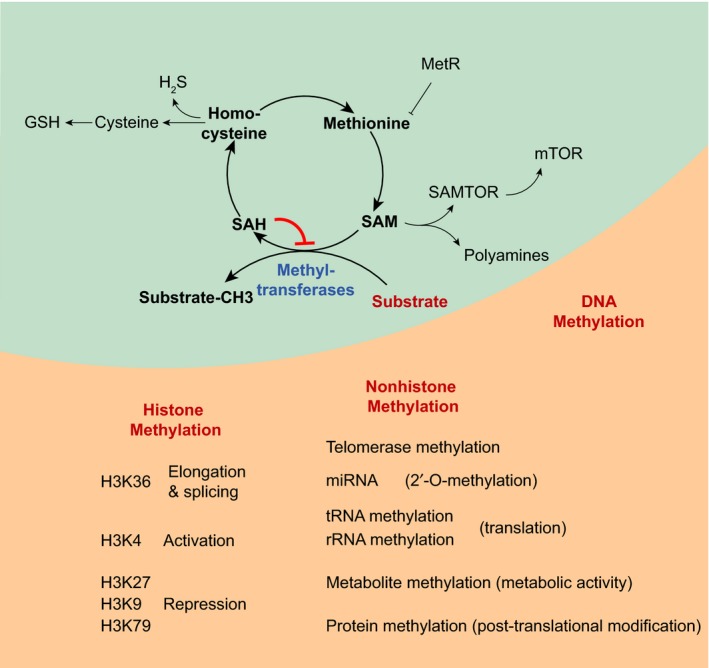
Methionine metabolism and methyltransferases

**Figure 3:**
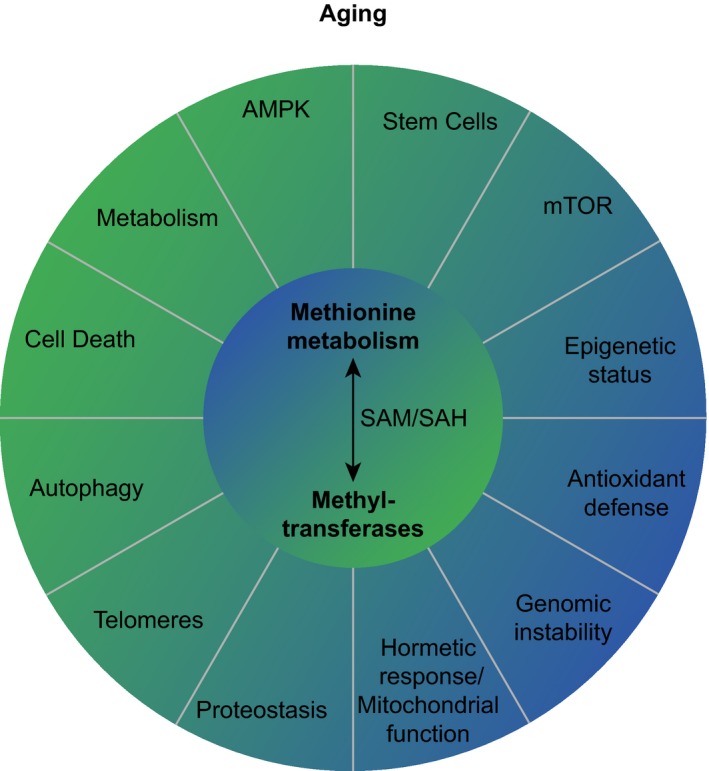
Mechanisms of lifespan extension by methionine metabolism and methyltransferases

In the following sections, we discuss how the different branches of methionine metabolism—the methionine cycle, the transsulfuration pathway, and polyamine metabolism—regulate lifespan (Figure 2) and discuss a potential mechanism linking methionine flux and lifespan regulation. We also review mechanisms of lifespan extension by MetR (Figure 3 and Table 1) with a main focus on the relevance of methionine metabolism and methyltransferases (Table 2) to lifespan extension. It should be noted that the mechanisms underlying lifespan extension by MetR and reprogramming of methionine metabolism (e.g., via activation of methionine metabolism flux) may overlap but are not identical. For example, both MetR and increasing SAH clearance as a result of the activation of flux via methionine metabolism can extend lifespan by impacting SAM/SAH level. However, as we discuss in this review, the methyltransferases affected by these two interventions and responsible for their lifespan phenotypes may differ.

**Table 1:** Manipulations of methionine metabolism that affect lifespan

Gene/metabolite	Manipulation	Description	Effect on lifespan	References
Yeast (*Saccharomyces cerevisiae*)				
Methionine	Restriction	Methionine restriction from the culture media	CLS extension	Johnson and Johnson (2014), Ruckenstuhl et al. (2014), Wu et al. (2013)
*met15*	Deletion	Limited endogenous methionine biosynthesis	CLS extension	Johnson and Johnson (2014), Ruckenstuhl et al. (2014)
*met2*	Deletion	No endogenous methionine biosynthesis	CLS extension	Johnson and Johnson (2014), Ruckenstuhl et al. (2014)
*met3*	Deletion	Sulfate assimilation and synthesis of methionine	RLS extension	McCormick et al. (2015)
*sam1*	Deletion	Methionine adenosyltransferase	RLS extension	McCormick et al. (2015)
*sam1*	Overexpression	Methionine adenosyltransferase	CLS extension	Ogawa et al. (2016)
*sam2*	Overexpression	Methionine adenosyltransferase	CLS extension	Ogawa et al. (2016)
SAH	Supplementation	*S*‐adenosylhomocysteine supplementation	CLS extension	Ogawa et al. (2016)
*cys4*	Deletion	Cystathionine beta‐synthase	CLS extension	Laschober et al. (2010)
NaHS	Supplementation	H_2_S donor	CLS extension	Hine et al. (2015)
GYY4137	Supplementation	H_2_S donor	CLS extension	Hine et al. (2015)
Spermidine	Supplementation	Polyamine	CLS and RLS extension	Eisenberg et al. (2009)
Worms (*Caenorhabditis elegans*)				
*Sams‐1*	Downregulation	Methionine adenosyltransferase	LS extension	Hansen et al. (2005)
Metformin	Supplementation	Metformin inhibits bacterial folate and methionine metabolism	LS extension	Cabreiro et al. (2013)
*anmt*‐1	Overexpression	Lowers level of SAM	LS extension	Schmeisser and Parker (2018)
*cbs‐1*	Overexpression	Cystathionine beta‐synthase	LS extension	Hine et al. (2015)
NAC	Supplementation	*N*‐acetyl‐l‐cysteine supplementation	LS extension	Oh et al. (2015)
Spermidine	Supplementation	Polyamine	LS extension	Eisenberg et al. (2009)
Flies (*Drosophila melanogaster*)				
Methionine + Amino acids	Restriction	Methionine restriction with reduced levels of amino acids	LS extension	Lee et al. (2014)
*dAhcyL1*	Downregulation	AHCY‐like protein 1	LS extension	Parkhitko et al. (2016)
*dAhcyL2*	Downregulation	AHCY‐like protein 2	LS extension	Parkhitko et al. (2016)
*Sams*	Downregulation	Methionine adenosyltransferase	LS suppression	Obata and Miura (2015)
*dCbs*	Overexpression	Cystathionine beta‐synthase	LS extension	Kabil et al. (2011)
*Gclc*	Overexpression	Glutamate–cysteine ligase, catalytic subunit	LS extension	Orr et al. (2005)
*Gclm*	Overexpression	Glutamate–cysteine ligase, modulatory subunit	LS extension	Orr et al. (2005)
NAC	Supplementation	*N*‐acetyl‐l‐cysteine supplementation	LS extension	Brack et al. (1997)
Spermidine	Supplementation	Polyamine	LS extension	Eisenberg et al. (2009)
Mammals				
Methionine	Restriction	Methionine restriction in rats, mice, and human cells	LS extension	Koziel et al. (2014), Miller et al. (2005), Orentreich et al. (1993)

**Table 2:** Manipulations of methyltransferases that affect lifespan

Gene/metabolite	Manipulation	Description	Effect on lifespan	References
Yeast (*Saccharomyces cerevisiae*)				
*trm9*	Deletion	tRNA methyltransferase	CLS extension	Fabrizio et al. (2010)
*rcm1*/*nsun5*	Deletion	rRNA cytosine methylase	CLS extension	Schosserer et al. (2015)
*tgs1*	Deletion	m_3_G cap formation of TLC1, RNA moiety of telomerase	RLS suppression	Franke et al. (2008)
*set2*	Deletion	H3K36‐specific methyltransferase	RLS extension	Ryu et al. (2014)
*set1*	Deletion	H3K4‐specific methyltransferase	RLS suppression	Cruz et al. (2018), Ryu et al. (2014)
*dot1*	Deletion	H3K79‐specific methyltransferase	RLS suppression	Ryu et al. (2014)
Worms (*Caenorhabditis elegans*)				
*anmt‐1*	Overexpression	Nicotinamide *N*‐methyltransferase	LS extension	Schmeisser et al. (2013), Schmeisser and Parker (2018)
*set‐9*	Downregulation	SET‐domain‐containing methyltransferase	LS extension	Greer et al. (2010), Hamilton et al. (2005), Ni et al. (2012)
*set‐15*	Downregulation	SET‐domain‐containing methyltransferase	LS extension	Hamilton et al. (2005)
*cec‐3*	Downregulation	H3K9 methyltransferase	LS extension	Greer et al. (2010), Hamilton et al. (2005)
*set‐4*	Downregulation	H3K20 methyltransferase	LS extension	Greer et al. (2010)
*set‐2*	Downregulation	H3K4 methyltransferase	LS extension	Greer et al. (2010)
*dot‐1.1*	Downregulation	H3K79 methyltransferase	LS extension	Wilhelm et al. (2017)
*mes‐2*	Downregulation	PcG protein, H3K27 methylation	LS extension	Ni et al. (2012)
*met‐1*	Downregulation	H3K36 methyltransferase	LS decrease	Pu et al. (2015)
*set‐18*	Downregulation	H3K36 methyltransferase	LS extension	Su et al. (2018)
*prmt‐1*	Overexpression	asymmetric arginine methyltransferase	LS extension	Takahashi et al. (2011)
*nsun‐5*	Downregulation	rRNA methyltransferase	LS extension	Schosserer et al. (2015)
Flies (*Drosophila melanogaster*)				
Gnmt	Overexpression	Glycine *N*‐methyltransferase	LS extension	Obata and Miura (2015)
PRC1	Downregulation	H3K27 trimethyltransferase complex	LS extension	Ma et al. (2018)
PRC2	Downregulation	H3K27 trimethyltransferase complex	LS extension	Ma et al. (2018), Siebold et al. (2010)
*pcmt*	Overexpression	Protein carboxyl methyltransferase	LS extension	Chavous et al. (2001)
*hen1*	Downregulation	2’‐O‐methylation	LS suppression	Abe et al. (2014)
*dNsun5*	Downregulation	rRNA methyltransferase	LS extension	Schosserer et al. (2015)
*dDnmt2*	Overexpression	tRNA methyltransferase	LS extension	Lin et al. (2005)
Mammals				
*Pcmt1*	Deletion	Protein carboxyl methyltransferase	LS suppression	Lowenson et al. (2001)
*Dnmt3a*	Deletion	de novo DNA methyltransferase 3a	LS suppression	Nguyen et al. (2007)
*Nrmt1*	Deletion	N‐terminal methyltransferase	LS suppression	Bonsignore et al. (2015)

## 2 METHIONINE METABOLISM AND LIFESPAN EXTENSION IN YEAST

The budding yeast *Saccharomyces cerevisiae* serves as one of the main model organisms for studying evolutionarily conserved mechanisms of aging and age‐related diseases. There are two aging models in budding yeast: chronological aging (CLS) and replicative aging (RLS). CLS is defined as the length of time that a nondividing yeast cell survives. RLS is defined as the number of daughter cells produced by a mother cell prior to senescence (Longo, Shadel, Kaeberlein, & Kennedy, 2012).

### 2.1 Methionine cycle

Wu et al. tested the effects of amino acids on CLS extension in the *S. cerevisiae* wild‐type BY4742 strain, which is auxotrophic for his/leu/lys. They found that changing the ratio of nonessential and essential amino acids caused great changes in CLS, whereas increase or decrease in individual amino acids in the culture media had little effect. The exceptions were methionine restriction and addition of glutamic acid, which resulted in CLS extension (Wu, Song, Liu, & Huang, 2013). Methionine restriction can also be achieved by genetically limiting methionine biosynthesis. Yeast can use inorganic sulfur to produce sulfur‐containing amino acids. To genetically induce MetR, Ruckenstuhl et al. and Johnson et al. used methionine‐auxotroph strains (genetic MetR) with limited (Δmet15) or no (Δmet2) endogenous methionine biosynthesis. These strains displayed enhanced CLS when grown on complete synthetic medium (i.e., with 30 mg/L methionine). In addition, restricting methionine from the medium increased CLS of the mutant strains but not of the prototrophic strain (Johnson & Johnson, 2014; Ruckenstuhl et al., 2014). Lifespan extension is often accompanied and dependent on TOR inhibition and activation of autophagy (Lopez‐Otin, Blasco, Partridge, Serrano, & Kroemer, 2013; Parkhitko, Favorova, & Henske, 2013). In yeast, MetR is accompanied by activation of autophagy flux, and deletion of autophagy essential genes or decrease in vacuolar acidity via disruption of v‐ATPase activity (which is responsible for the maintaining lysosomal pH) abolished MetR‐induced CLS extension. Moreover, MetR‐induced CLS extension could not be further extended by TOR inhibition, suggesting that MetR and TOR inhibition function via at least partially overlapping mechanisms (Ruckenstuhl et al., 2014). In various organisms including yeast, nuclear–mitochondrial communication is a key player in aging. Induction of the retrograde response pathway, which transmits signals of mitochondrial stress to the nucleus, can increase replicative lifespan in yeast (Kirchman, Kim, Lai, & Jazwinski, 1999). MetR‐induced CLS extension is dependent on the key mediator of retrograde signaling, the transcription factor *RTG3*, and 20% of differentially expressed genes under genetic MetR are reversed to basal levels in the absence of *RTG3* (Johnson & Johnson, 2014).

Interventions that extend lifespan often confer resistance to different stresses. In agreement with this, genetic MetR makes yeast cells more resistant to oxidative damage, heavy metal stresses (Singh & Sherman, 1974), heat stress, and lack of divalent metal cations, which is highly toxic to yeast cells (Johnson & Johnson, 2014). Unbiased genetic screening of 4,698 viable single‐gene deletion strains for replicative lifespan in *S. cerevisiae* identified that deletion of *MET3,* which encodes an enzyme important for the sulfate assimilation and synthesis of methionine, or *SAM1,* which encodes methionine adenosyltransferase, significantly extend replicative lifespan (McCormick et al., 2015), in agreement with inhibition of endogenous methionine biosynthesis in Δ*met15* and Δ*met2* mutant strains. In contrast to previous findings, however, Ogawa et al. found that stimulation of SAM synthesis by overexpression of *SAM1* or *SAM2,* or by external supplementation of SAH but not SAM, significantly extended CLS via Snf (AMPK) activation and *sah‐1* mutant had a shortened CLS (Ogawa et al., 2016).

### 2.2 Histone methylation

Imbalance in SAM/SAH ratio alters histone methylation. Yeast *cho2Δ* mutant cells, that have increased SAM/SAH ratios, have striking increases of the tri‐methylation H3K4me3, H3K36me3, and H3K79me3 marks (Ye, Sutter, Wang, Kuang, & Tu, 2017). The histone H3/H4 systematic mutant library is a versatile library of histone H3 and H4 mutants in which each residue has been substituted with alanine and alanine residues were substituted with serine. To test the importance of histone methylation to lifespan extension, Sen et al. (2015) screened this library and found that mutation of H3K36, a histone mark associated with transcriptional elongation and splicing, resulted in shorter replicative lifespan, whereas deletion of the only H3K36me3 demethylase, *Rph1*, extended lifespan. In contrast, Ryu et al. found that the replicative lifespan of *set2*‐deficient (H3K36‐specific methyltransferase) cells was significantly extended, whereas the replicative lifespans of *set1*‐deficient (H3K4‐specific methyltransferase) and *dot1* (H3K79‐specific methyltransferase)‐deficient cells were significantly shortened (Ryu, Rhie, & Ahn, 2014). H3K4me3 is primarily deposited by the COMPASS complex. *S. cerevisiae* has a single COMPASS complex containing the catalytic SET‐domain protein Set1 and core structural proteins Swd1, Swd3, Sdc1, Bre2, Swd2, and Spp1. Consistent with the previous report, Cruz et al. found that catalytic‐inactive *set1* ^H1017L^, Δ*swd1,* and Δ*spp1* mutant yeast strains exhibit significant RLS defect resulting specifically from loss of H3K4me3 (Cruz et al., 2018).

### 2.3 Nonhistone methylation

In light of the importance of methionine metabolism as a donor of SAM, growth in methionine‐deficient media causes tRNA hypomethylation (Fesneau, Robichon‐Szulmajster, Fradin, & Feldmann, 1975). Johnson and Johnson (2014) showed that genetic MetR via tRNA accumulation inhibits cytochrome C function and activates the retrograde response, which is required for MetR‐induced CLS extension. Genome‐wide studies and hypothesis‐driven approaches have identified several specific methyltransferases important for extension of yeast lifespan. A screen of 4,800 viable deletion mutants in *S. cerevisiae* (mentioned above) identified several processes including tRNA methylation that modulates yeast CLS. Deletion of *TRM9*, encoding a tRNA methyltransferase that methylates uridine residues at the wobble position in tRNA(Glu), tRNA(Gly), tRNA(Lys), tRNA(Gln), and tRNA(Arg3), almost tripled yeast mean CLS under starvation/extreme CR and increased heat resistance, but reduced resistance to acetic acid (Table 2). Modifications in the anticodon loops of tRNAs determine the rate of translation, as well as the accuracy and fidelity of translation. However, how deletion of *TRM9* interacts with MetR is not known (Fabrizio et al., 2010). Interestingly, Laxman et al. showed that the level of tRNA uridine thiolation reflects sulfur‐containing amino acid availability and connects translational capacity with amino acid homeostasis. Decreased availability of cysteine and methionine causes decreased tRNA uridine thiolation that suppresses translation and cell growth. Thiolation‐deficient yeast strains (Δ*uba4* and Δ*urm1*) and *mcm5*‐deficient strains (Δ*elp3* and Δ*trm9*) have increased CLS compared to controls. Uridine thiolation might represent the critical connection point between methionine metabolism activity, growth, and longevity (Laxman et al., 2013). Schosserer et al. found that *RCM1/Nsnu5* (rRNA cytosine methylase) was downregulated in chronologically aged yeast cells and CLS in *rcm1*‐knockout cells was significantly increased (although replicative lifespan was reduced). In addition, these *rcm1*‐knockout yeast cells were resistant to H_2_O_2_ (Schosserer et al., 2015).

*Saccharomyces cerevisiae* telomerase consists of three protein subunits, Est1, Est2, and Est3, and an RNA moiety, TLC1. TLC1 contains a methyl‐2,2,7‐guanosine (m_3_G or TMG) cap at its 5′ end. Franke et al. demonstrated that m_3_G cap formation of TLC1 depends on the methyltransferase Tgs1 and that this TLC1 modification influences telomere length and structure. Moreover, cells lacking Tgs1 activity exhibited premature aging (decreased replicative lifespan) (Franke, Gehlen, & Ehrenhofer‐Murray, 2008). It would be interesting to test how MetR affects the activity of Tgs1 methyltransferase and telomere length.

### 2.4 Transsulfuration pathway

The transsulfuration pathway allows interconversion of homocysteine and cysteine via the intermediary formation of cystathionine. *S. cerevisiae* possess two active transsulfuration pathways (Thomas & Surdin‐Kerjan, 1997), but mammalian cells can only convert homocysteine to cysteine. Laschober et al. identified gene expression signatures of cellular aging that are conserved between different human tissues and tested potential candidate genes for CLS extension in yeast. They found that deletion of CYS4, which encodes cystathionine beta‐synthase (CBS) or PDX3 (Pyridoxine [pyridoxamine] phosphate oxidase), which catalyzes the rate‐limiting step in the synthesis of pyridoxal 5′‐phosphate (vitamin B6), significantly extended CLS (Laschober et al., 2010). Dietary restriction in yeast via reduction of glucose in growth media promotes lifespan and increases H_2_S production via the transsulfuration pathway. In agreement with the beneficial role of H_2_S, the addition of exogenous H_2_S donors NaHS and GYY4137 significantly increased CLS (Hine et al., 2015).

### 2.5 Polyamine metabolism

Methionine metabolism is critical for the production of polyamines because it supplies an aminopropyl group necessary to the process (Minois et al., 2011; Pegg, 2016). Levels of the naturally occurring polyamine spermidine decrease with age. Eisenberg et al. showed that exogenous supplementation of spermidine led to a stable increase in intracellular spermidine levels in aging yeast cells and furthermore increased chronological and replicative yeast lifespans. Similar to genetic MetR, this lifespan extension was dependent on induction of autophagy, as deletion of *ATG7* (autophagy essential gene) compromised the lifespan‐extending effects of spermidine supplementation. Moreover, Eisenberg et al. (2009) identified that polyamine supplementation required suppression of histone acetylation and the double mutant for IKI3, which encodes an essential subunit of the histone acetylating elongator complex, and the histone acetyltransferase SAS3, increased the lifespan of yeast cells and abrogated the lifespan‐extending effects of spermidine. Similarly, McCormick et al. (2015) found in the above‐mentioned screen that deletion of *SAS3* significantly extends the yeast replicative lifespan.

Nutritional and genetic studies in yeast have identified roles for different branches of methionine metabolism in lifespan regulation, as well as identified specific relevant methyltransferases. Nevertheless, little is known about the crosstalk among various methionine‐relevant processes and how the crosstalk might impact lifespan.

## 3 METHIONINE METABOLISM AND LIFESPAN EXTENSION IN *CAENORHABDITIS ELEGANS*

### 3.1 Methionine cycle

In an unbiased RNAi screen, Hansen et al. identified 23 new longevity genes in *C. elegans* that when knocked down, extend lifespan from 10% to 90% (Hansen, Hsu, Dillin, & Kenyon, 2005). One of these genes, *sams‐1/*C49F5.1 encodes methionine adenosyltransferase, which catalyzes the biosynthesis of SAM, the first step in methionine metabolism. In *C. elegans*, the *eat‐2* mutant serves as a genetic model for studying DR, as mutation of *eat‐2* defects disrupts pharyngeal pumping and thus limits food intake. Hansen et al. found that knockdown of *sams‐1* extended lifespan in a *daf‐16*‐independent manner but failed to extend the lifespan of *eat‐2/ad1116* mutants. Knockdown of *sams‐1* did not affect pharyngeal pumping but similar to DR, knockdown of *sams‐1* resulted in slender worms with reduced brood size and delayed reproduction. Moreover, the level of *sams‐1* mRNA is reduced threefold in *eat‐2* mutants (Hansen et al., 2005). Ching, Paal, Mehta, Zhong, and Hsu (2010) showed that overexpression of *sams‐1* partially suppresses lifespan extension of DR worms and RNAi knockdown of *sams‐1* reduces the global translation rate. Lifespan extension via reduced activity of the first enzyme in the methionine cycle suggests that limiting flux through the pathway is beneficial for lifespan, preventing accumulation of harmful metabolites, and is consistent with the beneficial effects observed for MetR and DR. Consistent with this, metformin, a drug widely prescribed to treat type 2 diabetes, increases lifespan in *C. elegans* co‐cultured with *E. coli* as a food source after treatment with metformin. Metformin inhibits folate production and methionine metabolism in the bacteria, leading to changes in methionine metabolism and a decrease in the level of SAM in the worms (Cabreiro et al., 2013).

Sirtuins are a family of histone deacetylases that require NAD^+^ as a cosubstrate. During sirtuin‐mediated deacetylation of l‐lysine residues, NAD^+^ is converted into nicotinamide (NAM), which can be methylated by nicotinamide *N*‐methyltransferase (encoded by *anmt‐1* in *C. elegans*) to 1‐methylnicotinamide (MNA). Schmeisser et al. (2013) showed that ANMT‐1 overexpression extends *C. elegans* lifespan. Later, Schmeisser et al. showed that ANMT‐1 competes for SAM with the methyltransferase leucine carboxyl methyltransferase 1 (LCMT‐1), which is known to regulate autophagy. High NNMT activity leads to low SAM levels (similar to DR), serving as a starvation signal and thereby inducing autophagy. In addition, expression of ANMT‐1 only in dopaminergic neurons suppressed age‐dependent neurodegeneration and extended lifespan and was dependent on neuronal autophagy (Schmeisser & Parker, 2018). Moreover, Liu et al. (2019) showed that supplementation with glycine significantly prolongs *C. elegans* lifespan via feeding into the methionine cycle and that mutations in components of this cycle, methionine synthase (*metr‐1*) and *S*‐adenosylmethionine synthetase (*sams‐1*), completely abrogate glycine‐induced lifespan extension.

### 3.2 Histone methylation

In a large‐scale RNAi screen, Hamilton et al. identified 89 new genes that extend lifespan in *C. elegans*. Three of these genes, *F15E6.1/Set‐9*, *R11E3.4/Set‐15,* and T09A5.8/cec‐3, are predicted to encode proteins that contain a SET domain, a signature motif of most histone methyltransferases (Hamilton et al., 2005).

### 3.3 H3K4 (activation histone mark)

The methylation state of H3K4 strongly correlates with transcriptional activation, and methylation status at H3K4 depends on the activity of histone lysine methyltransferases (KMTs), histone lysine demethylases (KDMs), and can be affected by the activity of methionine metabolism. *C. elegans* with reduced function of *sams‐1* and concomitant decrease in SAM have a broad decrease in the global H3K4me3 levels (Ding et al., 2015). KDM1 (in worms, KDM1A—*spr‐5* and *lsd‐1*, KDM1B—*amx‐1*) removes only Me1 and Me2 from H3K4, and KDM5 (*rbr‐2* is a sole KDM5 member in *C. elegans*) can remove Me3 and Me2 from H3K4. Greer et al. performed a targeted RNAi screen by selecting genes that encode known worm methyltransferases and identified that knockdown of *set‐4, set‐9, set‐15,* and members of ASH‐2 trithorax complex (ASH‐2, WDR‐5, and SET‐2), which trimethylates histone H3 at lysine 4 (H3K4), extended *C. elegans* lifespan. Further, overexpression of RBR‐2 (H3K4me3 demethylase that opposes the activity of ASH‐2 trithorax complex by removing methyl marks) extends lifespan (Greer et al., 2010). Strikingly, the effect of ASH‐2, WDR‐5, or SET‐2 depletion on lifespan is inherited through several generations (wild‐type F3 and F4 descendants from *ash‐2, wdr‐5,* or *set‐2* mutants were long‐lived) (Greer et al., 2011). Surprisingly, deletion of H3K4me2 demethylase, *spr‐5*, also causes a trans‐generational increase in lifespan. The lifespan extension induced by disruption of the H3K4 trimethylase complex occurs immediately after deletion of H3K4 trimethylase complex and disappears after three generations. Different from this, deletion of *spr‐5* initially has no observable effect but a longevity extension appears after seven generations (Greer, Becker, Latza, Antebi, & Shi, 2016). McColl et al. (2008) found that exposure to lithium throughout adulthood increased longevity via a sharp reduction of expression of H3K4 histone demethylase LSD1 (KDM1A) and that knockdown of LSD1 is sufficient to extend lifespan. In agreement with these observations, Alvares et al. found that a *rbr‐2* mutant strain has a longer lifespan, and both *rbr‐2* and *spr‐5* are required for extended longevity in daf‐2 adults, suggesting their anti‐aging function (Alvares, Mayberry, Joyner, Lakowski, & Ahmed, 2014). Further, Wilhelm et al. performed a screen for genes that extend postreproductive lifespan using an RNAi library targeting 800 factors involved in chromatin and transcriptional regulation. Among the 36 longevity genes identified in the screen were *rbr‐2* (H3K4me3 demethylase) and *dot‐1.1* (H3K79‐specific methyltransferase) (Wilhelm et al., 2017). However, there is conflicting evidence with regard to the effects of up‐ or downregulation of H3K4 methylation on lifespan extension, as downregulation of either H3K4 methyltransferase complex or demethylase can prolong lifespan. H3K4 methylation might be a potential mechanistic target for MetR, as this is among the most sensitive histone methylation site that is suppressed under MetR (Mentch et al., 2015).

### 3.4 H3K27 (repressive histone mark)

Jin et al. observed increased activity of the H3K27 demethylase UTX‐1 in *C. elegans* during aging and corresponding loss of H3K27me3 on the regions corresponding insulin/IGF‐1 signaling pathway components. RNAi knockdown of UTX‐1, which extends the lifespan of *C. elegans* by 30%, does not have an additive effect when combined with mutation of *daf2*, and increases H3K27me3 on the *Igf1r/daf‐2* gene (Jin et al., 2011). In a targeted RNAi screen focused on histone demethylases, Maures et al. identified four histone demethylases that regulate *C. elegans* lifespan, UTX‐1 (KDM6A, H3K27me2/me3 demethylases), RBR‐2 (JARID1/KDM5B; H3K4me2/me3 demethylases), LSD‐1 (KDM1A; H3K4me1/me2, H3K9me2 demethylases), and T26A5.5 (JHDM1B/KDM2B, H3K36me2 demethylases). Moreover, Ni, Ebata, Alipanahiramandi, and Lee (2012) performed a small‐scale RNAi screen targeting most putative histone methyltransferases and demethylases in worms and identified six longevity genes that, when inactivated by RNAi, can extend worm lifespan: *set‐9/26* (MLL5), *mes‐2* (E(Z)), *utx‐1* (UTX), *jmjd‐2* (KDM4), and *rbr‐2* (JARID1). Four of these six genes, *set‐9, set‐26* (H3K9 trimethyltransferase)*, rbr‐2,* and *utx‐1,* were previously identified as longevity‐associated genes in worms. In contrast to lifespan extension by UTX‐1 downregulation, overexpression of another H3K27me3 demethylase, JMJD‐3.1, extended lifespan (Labbadia & Morimoto, 2015). Overexpression of the H3K9 and H3K27me2/3 demethylase jmjd‐1.2, but not of the catalytically inactive jmjd‐1.2^H508A^, led to significantly increased longevity, whereas downregulation of *jmjd‐1.2* and *jmjd‐3.1* suppressed ETC‐mediated longevity in *C. elegans* (Merkwirth et al., 2016).

### 3.5 H3K36 (histone mark, associated with transcriptional elongation and splicing)

Deletion of met‐1 H3K36 methyltransferase decreased lifespan in *C. elegans* and increased the level of mRNA expression noise with age (Pu et al., 2015). Su et al. compared expression of known histone methyltransferases and proteins containing the SET enzymatic domain of methyltransferases in young and old *C. elegans* and found that the levels of *set‐2*, *set‐10*, *set‐18*, *set‐32*, and *F54F7.7* were upregulated in old worms. They further demonstrated that like downregulation of the known lifespan regulator *set‐2*, downregulation of *set‐18* also led to extended lifespan, and identified set‐18 as a histone H3K36 dimethyltransferase. They demonstrated that SET‐18 represses *daf16a* promoter through H3K36me2 modification and muscle‐specific expression of SET‐18 in old worms was responsible for shortening lifespan (Su et al., 2018).

### 3.6 Nonhistone methylation

Takahashi et al. (2011) studied arginine methylation, a posttranslational protein modification, and identified PRMT‐1 as the major asymmetric arginine methyltransferase in *C. elegans*. They demonstrated that PRMT1 methylates DAF‐16, thereby blocking its phosphorylation by AKT. In addition, loss of *prmt‐1* shortened lifespan and overexpression of PRMT1 significantly increased lifespan when co‐overexpressed with DAF‐16.

In yeast, *rcm1*‐knockout cells have an increased replicative lifespan. In *C. elegans*, depletion of *RCM1*/*nsun‐5* (25S rRNA cytosine 2278‐specific methylase) by RNAi extended the lifespan of worms with complete removal of food during adulthood or in *eat‐2* mutant worms, but the effect was completely abrogated in worms fed ad libitum (Schosserer et al., 2015).

### 3.7 Transsulfuration pathway

As noted above, in *C. elegans*, the *eat‐2* mutant strain serves as a genetic model for studying DR. *Eat‐2* mutant worms produced more H_2_S, a product of the transsulfuration pathway, than wild‐type worms. CBS‐1 is a worm ortholog of cystathionine‐β‐synthase, the rate‐limiting enzyme in the transsulfuration pathway (Figure 1). RNAi knockdown of *cbs‐1* decreased the lifespan extension normally associated with *eat‐2* mutants, and overexpression of CBS‐1 in wild‐type worms prolonged lifespan (Hine et al., 2015). Supplementation of the diet with the product of the transsulfuration pathway, *N*‐acetyl‐l‐cysteine, significantly extended both the mean and maximum lifespan and significantly increased resistance to oxidative stress, heat stress, and UV irradiation in *C. elegans* (Oh, Park, & Park, 2015).

### 3.8 Polyamine metabolism

Similar to yeast, supplementation of the food with spermidine in *C. elegans* induced autophagy and prolonged lifespan by up to 15%, whereas knockdown of *Beclin‐1*, a gene essential for autophagy, abolished the spermidine‐mediated increase in lifespan (Eisenberg et al., 2009).

## 4 METHIONINE METABOLISM AND LIFESPAN EXTENSION IN *DROSOPHILA MELANOGASTER*

### 4.1 Methionine cycle

DR is one of the most effective dietary interventions that extends lifespan in diverse organisms (Tatar, Post, & Yu, 2014), but also leads to reduced fecundity. Methionine supplementation alone can increase fecundity in DR animals to levels comparable to full‐fed controls without reducing lifespan (Grandison, Piper, & Partridge, 2009). Several groups have developed semi‐ or fully defined fly diets for testing the effects of specific food components on lifespan. The basal level of methionine significantly varies in these diets, ranging from 0.34 to 1.65 g/L (Piper, 2017). To test the effect of MetR in flies and its interaction with DR, Lee et al. compared lifespans of flies using diets with different levels of amino acids and different concentrations of methionine. Restriction of amino acids increased lifespan at any concentration of methionine, whereas MetR extended lifespan only in conditions when the levels of amino acids were reduced (Lee et al., 2014). Interestingly, we recently showed that naturally selected long‐lived flies, which have twice the lifespan of wild‐type strains, have higher levels of endogenous methionine, suggesting that high levels of methionine are not detrimental to lifespan and that flux via methionine metabolism is more critical than the level of methionine itself (Parkhitko et al., 2016). In agreement with this hypothesis, we suggest that methionine metabolism is reprogrammed during aging that leads to the accumulation of SAH and homocysteine. Downregulation of *dAhcyL1/dAhcyL2* at the whole‐organism and tissue‐specific level extends lifespan and healthspan. In our model, downregulation of *dAhcyL1/dAhcyL2* activates Ahcy13, which in turn promotes SAH and homocysteine processing, resulting in an increase in methionine flux and an effect reminiscent of methionine restriction (Parkhitko et al., 2016).

In agreement with the importance of flux via methionine metabolism, Obata et al. showed that overexpression of GNMT, which converts glycine to sarcosine (*N*‐methyl‐glycine) by methyl group transfer using SAM and functions as a regulator of SAM levels in metabolic organs, suppresses age‐dependent SAM increase and extends lifespan (Obata & Miura, 2015). Interestingly, this group also found that, in contrast to *C. elegans* where *sams‐1/*C49F5.1 (methionine adenosyltransferase, catalyzes the biosynthesis of SAM, the first step in methionine metabolism) extended lifespan, knockdown of *Sams* in flies significantly shortened lifespan. Notably, there are four *Sams* genes in *C. elegans* but only one in *Drosophila,* such that suppression of *Sams* in *Drosophila*, but not in *C. elegans,* should completely block the methionine cycle. Interestingly, tissue‐specific activation of methionine metabolism flux via downregulation of *dAhcyL1/L2* in the brain or intestine extends healthspan and lifespan (Parkhitko et al., 2016)*.* In accordance, Obata et al. (2018) identified SAM as a critical regulator of stem cell division in the *Drosophila* midgut, acting via regulation of *Dph5*, *HemK1*, and *HemK2* methyltransferases and control of protein translation. Based on this, we suggest that tissue‐specific dysregulation of methionine metabolism can be a driving force of age‐dependent intestinal dysplasia and further, that either MetR or activation of methionine flux can prevent these changes, improve proliferative homeostasis, and extend lifespan. Furthermore, Kashio et al. (2016) found that alterations of SAM metabolism in the fat body via up‐ or downregulation of GNMT affect repair of wing disks, showing that organ‐specific changes in methionine metabolism have systemic effects on regenerative processes. It will be of interest to further understand how methionine metabolism is altered with age in different organs and create genetic tools that will allow dissection of the role(s) of methionine metabolism in specific tissues and organs.

Several histone and nonhistone methyltransferases in flies have been shown to regulate lifespan, as discussed below.

### 4.2 Histone methylation

#### 4.2.1 H3K4 (activation histone mark)

Liu et al. tested the effects on histone methylation of three core enzymes involved in methionine metabolism, Sam‐S, Ahcy13, and Cbs. They found that downregulation of *Sams* led to decreased levels of H3K4me3 and H3K9me2 in *Drosophila* S2 cells (Liu, Barnes, & Pile, 2015)*.* Consistent with this, we found that activation of flux in methionine metabolism via downregulation of *dAhcyL1* significantly suppressed the level of H3K4me3 (Parkhitko et al., 2016). The corepressor SIN3, which controls histone acetylation through association with the histone deacetylase RPD3, binds to the promoter regions of genes involved in methionine metabolism and regulates levels of SAM and H3K4me3 (Liu & Pile, 2017). Interestingly, Liu and Pile (2017) showed that SIN3 regulates crosstalk between histone acetylation and histone methylation via regulation of methionine metabolism.

#### 4.2.2 H3K27 (repressive histone mark)

The levels of H3K27 methylation have been shown to affect lifespan in *Drosophila*. Flies heterozygous for mutations in *E(z)* and *esc*, members of the PRC2 H3K27 trimethyltransferase complex, decreased levels of H3K27me3 and extended lifespan (Siebold et al., 2010). Ma et al. further confirmed this by demonstrating that levels of H3K27me3 increase with age in *Drosophila* and reduction of components of PRC2 (*esc*, *E(z)*, *Pcl*, *Su(z)12*), and PRC1 (*Psc* and *Su(z)2*) promote lifespan. Moreover, they showed that PRCs‐deficiency promotes glycolysis and increase in glycolytic genes in wild‐type animals extends longevity (Ma et al., 2018). As mentioned above, suppression of H3K27me3 demethylase (UTX‐1) in worms also extends lifespan (different from what is observed for flies); however, overexpression of H3K27me3 demethylase JMJD‐3.1 extended lifespan, suggesting that the genes regulated by H3K27me3 are organism‐ and tissue‐specific. Also in contrast to *C. elegans,* in which suppression of RBR‐2 results in elevated lifespan (Greer et al., 2010, 2011), Li, Greer, Eisenman, and Secombe (2010) showed that male flies mutant for *little imaginal disk (lid),* which encodes the *Drosophila* H3K4me3 demethylase, has a significantly shorter lifespan, and an effect that was not observed in females.

#### 4.2.3 H3K9 (repressive histone mark)

Whereas euchromatic regions (transcriptionally active) are characterized by increased levels of H3K4me3 and H3K36me3, and heterochromatic regions (transcriptionally silent) are characterized by having increased levels of H3K9me2 or 3, which are able to recruit heterochromatin protein‐1 (HP‐1) to advance heterochromatin formation (H3K9me2 can also mark inactive euchromatin). Aging is associated with loss of repressive heterochromatin marks and can be measured in *Drosophila* using a position effect variegated (PEV) LacZ reporter gene (Jiang et al., 2013), in which *LacZ* is inserted at the boundary between euchromatin and heterochromatin; the level of heterochromatization determines the level of *LacZ* expression. In *Drosophila*, specific heterochromatin regions are remodeled during aging and that lifespan‐extending interventions such as calorie restriction suppress age‐dependent heterochromatin remodeling (Jiang et al., 2013; Wood et al., 2010). Moreover, heterochromatin formation prolongs lifespan and heterochromatin formation depends on the levels of HP1 and H3K9 methylation (Larson et al., 2012). A significant decrease in both H3K4me3 and H3K36me3 was observed in older flies (Wood et al., 2010).

### 4.3 Nonhistone methylation

Accumulation of damaged (isoaspartyl‐containing) proteins has been demonstrated in different species including mice. The accumulation of these potentially dysfunctional isoaspartyl proteins is counteracted by a protein carboxyl methyltransferase (PCMT). Chavous et al. demonstrated that flies ubiquitously expressing PCMT have an increased lifespan at 29°C; however, lifespan extension was not observed at 25°C (Chavous, Jackson, & O'Connor, 2001). Abe et al. studied how microRNAs expression pattern changes with age. In *Drosophila*, most miRNAs are loaded into Ago1 and remain unmodified; however, some miRNAs are found to be 2′‐O‐methylated and loss of 2′‐O‐methylation of small RNAs leads to destabilization as well as tailing and trimming of small RNAs. In *Drosophila*, 2′‐O‐methylation of small RNAs depends on the methyltransferase Hen1 and is associated with loading of miRNAs into Ago2. Abe et al. found that age‐associated increase in specific *Drosophila* miRNAs isoforms reflected increased 2′‐O‐methylation and loading of these isoforms into Ago2. They also found that the lack of 29‐O‐methylation by *Hen1* and *Ago2* mutations resulted in reduced lifespan and brain degeneration (Abe et al., 2014). Similar to yeast and worms, ubiquitous downregulation of *RCM1*/*dNsun5* in male flies extended the lifespan of flies by 16%–20%, but the effect was abrogated on the diet that was richer in sugar and yeast. Overexpression of dNsun5 reduced mean lifespan by 58% (Schosserer et al., 2015). Lin, Tang, Reddy, and Shen (2005) demonstrated that overexpression of dDnmt2, a methyltransferase, can extend the *Drosophila* lifespan. *Drosophila* is thought to have no or low levels of genomic 5‐methylcytosine. The human homolog of dDnmt2, *TRDMT1*/DNMT2, does not display DNA methyltransferase activity but can methylate aspartic acid tRNA (Goll et al., 2006).

### 4.4 Transsulfuration pathway

Cystathionine β‐synthase (CBS) catalyzes the first and rate‐limiting step in the transsulfuration pathway, which involves pyridoxal 5′‐phosphate‐dependent condensation of serine and homocysteine to form cystathionine. Kabil et al. demonstrated that activity of dCBS and the transsulfuration pathway is increased under DR and inhibition of the second enzyme in transsulfuration pathway, γ‐cystathionase, using propargylglycine, causes robust suppression of lifespan extension by DR but not in fully fed flies. In agreement with this, either ubiquitous adult‐specific overexpression of dCBS or neuronal overexpression of dCBS is sufficient to increase longevity (Kabil, Kabil, Banerjee, Harshman, & Pletcher, 2011). In agreement with the positive role of the TSP in lifespan extension, maximal H_2_S production of flies subjected to various forms of Met and DR correlated with maximal lifespan extension (Hine et al., 2015). Cysteine is a product of the transsulfuration pathway and a precursor for the synthesis of tripeptide γ‐glutamylcysteinylglycine (glutathione, GSH). GSH helps maintain cellular redox homeostasis, acts as a xenobiotic conjugant, facilitating export of xenobiotics from cells, participates in thiolation and dethiolation of proteins, and has additional roles. Glutamate–cysteine ligase (GCL) is the rate‐limiting enzyme that conjugates glutamate and cysteine to create γ‐glutamylcysteine. GSH synthase (GS) links glycine to γ‐glutamylcysteine to form GSH. Glutamate–cysteine ligase (GCL) is a heterodimeric enzyme consisting of a catalytic subunit, GCLc, and a modulatory subunit, GCLm. Orr et al. (2005) showed that the overexpression of GCLc or GCLm in flies using either global or neuronal drivers of expression led to an increase in the glutathione content observed in fly homogenates and extended lifespan. In agreement with these findings, feeding flies with a cysteine donor for GSH, *N*‐acetylcysteine (NAC), results in a dose‐dependent increase in lifespan (Brack, Bechter‐Thuring, & Labuhn, 1997).

### 4.5 Polyamine metabolism

Similar to yeast and worms, supplementation in *Drosophila* of regular food with 1 mM spermidine was shown to prolong lifespan by up to 30% (Eisenberg et al., 2009).

## 5 METHIONINE METABOLISM AND LIFESPAN EXTENSION IN MICE, RATS, AND HUMAN CELLS

### 5.1 Methionine cycle

It was first shown in rats that MetR leads to an increase in lifespan. The lifelong reduction of a single dietary component, methionine, from 0.86% to 0.17% in Fisher 344 rats resulted in 30% increase in male rat lifespan (Orentreich et al., 1993). In female CB6F1 mice, decrease in methionine from 0.43% by weight to 0.1%–0.15% increased lifespan, slowed immune and lens aging, and decreased levels of serum IGF‐I, insulin, glucose, and thyroid hormone, despite an increase in food uptake (Miller et al., 2005). In mice, the ideal range of dietary methionine restriction is from 0.17% to 0.25% (Forney, Wanders, Stone, Pierse, & Gettys, 2017). Restriction of dietary methionine to levels above 0.25% was without effect while restriction to levels below 0.12% produced responses characteristic of essential amino acid deprivation. Although restriction of dietary methionine to 0.12% does not evoke essential amino acid deprivation responses, it provides insufficient methionine to support growth (Forney et al., 2017). In human diploid fibroblasts, reduction of methionine from 30 to 1 mg/L had no significant effect on the rate of cell proliferation in early passage cells but significantly extended their replicative lifespan, postponing cellular senescence. Extended lifespan was associated with reduced oxygen consumption (Koziel et al., 2014). In humans, an 83% reduction in daily methionine uptake for 3 weeks reduced plasma methionine levels and altered circulating metabolism with the most effect on cysteine and methionine metabolism (Gao et al., 2019). In addition, in humans, vegan diet is associated with decreased methionine content (McCarty, Barroso‐Aranda, & Contreras, 2009). In mice, gene expression profiles of MetR and CR mice do not significantly overlap, suggesting that these two dietary regimens affect longevity through partly independent pathways (Sun, Sadighi Akha, Miller, & Harper, 2009). MetR restores a younger metabolic phenotype in adult mice: MetR in 12‐month‐old mice (0.172% vs. 0.86% methionine) reversed age‐induced alterations in body weight, adiposity, physical activity, and glucose tolerance to levels observed in healthy 2‐month‐old control‐fed mice. MetR also causes lipid metabolism reprogramming in mice. Short‐term (48 hr) MetR increases hepatic fibroblast growth factor‐21 (FGF21) expression/secretion (Lees et al., 2014). In young animals, MetR stunts growth and development, reducing total length, levels of serum insulin‐like growth factor 1 (IGF‐1), and growth hormone signaling activity. FGF21 is an atypical FGF that is secreted by the liver during fasting and elicits diverse aspects of the adaptive starvation response. Multiple beneficial effects of FGF21 are similar to MetR. FGF21 induces hepatic fatty acid oxidation and ketogenesis, increases insulin sensitivity, blocks somatic growth, and causes bone loss. Transgenic mice overexpressing FGF21 are markedly smaller, have decreased circulating IGF‐1 concentrations, and have a significantly extended lifespan (Zhang et al., 2012). The multiple physiological outcomes of MetR were recently reviewed and will not be further discussed here (Ables, Hens, & Nichenametla, 2016; Cavuoto & Fenech, 2012; Lee, Kaya, & Gladyshev, 2016; McIsaac, Lewis, Gibney, & Buffenstein, 2016; Sanchez‐Roman & Barja, 2013; Zhou et al., 2016). Methionine metabolism is also altered in the tissues of long‐lived Ames mice (Uthus & Brown‐Borg, 2006) and naked mole‐rats (Ma et al., 2015); however, whether these changes are causative or correlative is not known. Interestingly, Gu et al. recently demonstrated that SAM disrupts the SAMTOR‐GATOR1 complex by binding directly to SAMTOR. GATOR1, the GTPase activating protein for RagA/B, promotes the localization of mTORC1 to the lysosomal surface, its site of activation. MetR reduces SAM levels and promotes the association of SAMTOR with GATOR1, thereby inhibiting mTORC1 signaling in a SAMTOR‐dependent fashion (Gu et al., 2017). Because mTORC1 is a central regulator of aging (Parkhitko, Favorova, Khabibullin, Anisimov, & Henske, 2014; Saxton & Sabatini, 2017), SAMTOR, a SAM sensor, might serve as a critical connection hub between methionine metabolism, mTORC1 signaling, and aging.

Another important player in the methionine cycle and aging is homocysteine. In human plasma, total homocysteine is present in four forms: 1%–2% as the thiol form, homocysteine; 82%–83% combined in disulfide linkage with cysteines of proteins (mostly albumin); and the remaining 15%–16% as the free disulfides homocysteine and cysteine–homocysteine disulfide (all these fractions are called total homocysteine, tHCY). One reason homocysteine can be harmful is because homocysteine can be converted to thiolactone as a result of an error‐editing function of some aminoacyl‐tRNA synthetases. Thiolactone is chemically reactive and acylates free amino groups in proteins. The amount of thiolactone formation is dependent on methionine flux (Jakubowski, Zhang, Bardeguez, & Aviv, 2000). A homocysteine thiolactonase, paraoxonase I, detoxifies thiolactone via hydrolysis. In addition, homocysteine can cause neuronal death serving as an agonist for metabotropic glutamate receptors as well as for NMDA and AMPA/kainite ionotropic glutamate receptors particularly via ERK activation (Poddar & Paul, 2009). Moreover, high levels of homocysteine upregulate mTORC1 activity. Homocysteine is sensed by protein complex composed of leucyl‐tRNA‐synthetase and folliculin, which regulates activity of mTORC1 via its tethering to lysosomal membranes (Khayati et al., 2017). Chronic age‐dependent inhibition of the flux via methionine metabolism would result in upregulated levels of homocysteine. One of the consequences of high level of homocysteine would be activation of mTORC1 and chronic inhibition of the molecular clearance of protein products. Interestingly, in both mice and rats MetR leads to hyperhomocysteinemia (Ables et al., 2015; Elshorbagy et al., 2010; Perrone et al., 2012; Tamanna, Mayengbam, House, & Treberg, 2018). Further studies are required to clarify why MetR extends lifespan and exerts multiple benefits while increasing the level of potentially harmful homocysteine.

### 5.2 DNA, histone, and nonhistone methylation

In general, aging is associated with DNA hypomethylation; however, some DNA regions become hypermethylated. In mammals, DNA methylation patterns change with age (Hannum et al., 2013) and can serve as a marker for chronological age (Horvath, 2013), but it is not known whether these changes are causative or correlative. As mentioned above, methionine metabolism is significantly altered in the tissues of long‐lived Ames mice (Uthus & Brown‐Borg, 2006). Similar to the expression of methionine metabolism genes, DNMTs (DNA methyltransferases) are also differentially expressed in Ames mice compared with wild‐type siblings, potentially creating a regulatory loop between methionine metabolism, DNA methylation and lifespan regulation (Armstrong, Rakoczy, Rojanathammanee, & Brown‐Borg, 2014). Moreover, age‐associated methylation changes are suppressed in longer‐lived Ames dwarf, calorie‐restricted and rapamycin‐treated mice (Cole et al., 2017; Wang et al., 2017). Mattocks et al. (2017) demonstrated that MetR improves the efficiency of DNA methylation maintenance systems in adult mice but had no effect in young mice and the effect of MetR on DNA methylation was tissue‐specific and was driven by changes in SAH. We hypothesize that the activity of methionine metabolism is directly sensed by DNA methyltransferases that set up the biological age reflected in epigenetics clocks. Based on this hypothesis, various lifespan‐extending regimens would reset the epigenetic clocks via altering the activity of methionine metabolism that in turn would determine the activity if DNA methyltransferases. A regulatory loop between DNA methylation and methionine metabolism has been described by Zhou et al. They showed that H19 lncRNA binds to and inhibits *S*‐adenosylhomocysteine hydrolase (SAHH) and that this interaction prevents SAHH from hydrolyzing SAH, leading to block of DNA methylation by DNMT3B at numerous genomic loci (Zhou et al., 2015). Mitochondrial dysfunction, which is commonly observed with aging, can also alter DNA methylation patterns via effects on methionine metabolism; namely, leading to an increase in the SAM/SAH ratio and an increase in DNA methylation (Lozoya et al., 2018).

In contrast to yeast, worms, and flies, for mice, information about specific methyltransferases capable of extending lifespan is limited. As discussed earlier, flies expressing PCMT have an extended lifespan (Chavous et al., 2001), whereas *Pcmt1−/−* mice die at a mean age of 42 days from seizures (Lowenson, Kim, Young, & Clarke, 2001). Depleting the H3K9me3 methyltransferase Suv39h1 in a progeria mouse model reduces H3K9me3 levels, delays senescence in progeroid cells, and extends lifespan, but whether perturbation of Suv39h1 can extend lifespan in healthy mice is not known (Liu et al., 2013). Mice lacking functional de novo DNA methyltransferase Dnmt3a are born healthy but degenerate in adulthood and die prematurely (Nguyen, Meletis, Fu, Jhaveri, & Jaenisch, 2007). Mice deficient for NRMT1, N‐terminal methyltransferase, which regulates protein‐DNA interactions, exhibit phenotypes associated with impaired DNA repair and premature aging (Bonsignore et al., 2015).

### 5.3 Transsulfuration pathway

Similar to worms and flies, a heterogeneous stock of mice (NIA Interventions Testing Program) fed with *N*‐acetyl‐l‐cysteine (NAC) had a significantly extended lifespan. Notably, the effect was sex‐specific. In females, NAC treatment did not significantly affect total lifespan. In males, both high (1,200 mg kg^−1^ day^−1^) and low (600 mg kg^−1^ day^−1^) NAC doses increased total lifespan. However, both doses of NAC caused a sudden drop in body weight, followed by a further slow decline (Flurkey, Astle, & Harrison, 2010). A characteristic of aging and a major cause of mortality and morbidity is the age‐dependent decline in capillary density and blood flow. Recently, two groups showed that H_2_S, a product of enzymes with roles in transsulfuration pathway, promotes angiogenesis (Das et al., 2018; Longchamp et al., 2018). Longchamp et al. demonstrated that MetR increased VEGF expression in vitro and increased capillary density in mouse skeletal muscle in vivo via the GCN2/ATF4 amino acid starvation response pathway. This effect was independent of hypoxia or HIF1α. Cystathionine‐γ‐lyase was required for VEGF‐dependent angiogenesis via increased production of H_2_S that mediated its proangiogenic effects in part by inhibiting mitochondrial electron transport chain activity (Longchamp et al., 2018). Das et al. demonstrated that increasing the expression of SIRT1 in endothelial cells is sufficient to increase the capillary density of skeletal muscle, enhance exercise performance, and increase VEGF serum protein levels. Administration of the NAD+ precursor, NMN, to 18‐month‐old mice restored the number of capillaries and capillary density of the old mice to the level in young mice. Moreover, they demonstrated that H_2_S similar to NMN increases the level of SIRT1 and the combination of NAD precursor, NMN, and H_2_S donor, NaHS, more potently increased capillary density and reduced the number of apoptotic ECs in vivo and physical endurance of mice than either treatment alone (Das et al., 2018). Interestingly, Tyshkovskiy et al. (2019) found that cystathionine‐γ‐lyase was one of the most significant commonly upregulated genes among 15 known lifespan‐extending interventions in mice.

### 5.4 Polyamine metabolism

Similar to yeast, worms, and flies, in C57BL/6J female mice, supplementation with spermidine and spermine (but not putrescine) significantly extends median lifespan. Spermidine also significantly extends lifespan when supplementation is started late in life (starting at 18‐month‐old mice). Spermidine supplementation has cardioprotective effects, resulting in reduced cardiac hypertrophy and preserved diastolic function in old mice. These organ‐level findings are associated with enhanced cardiac cell autophagy, mitophagy, and mitochondrial respiration and spermidine fails to provide cardioprotection in mice that lack Atg5, which is essential for autophagy in cardiomyocytes (Eisenberg et al., 2016). It will be of interest to determine how MetR affects the levels of polyamines in different tissues and whether the mechanisms of lifespan extension by MetR and supplementation with spermidine and spermine overlap. MetR can be expected to decrease the levels of spermidine and spermine since it decreases the level of dcSAM. However, MetR extends lifespan and causes a 10‐fold increase in the polyamine spermidine in livers of *Lmna* progeroid mice (Barcena et al., 2018).

## 6 CONCLUSION

Although the effect of MetR on lifespan extension was reported in 1993, the mechanisms at play are not completely understood. Our recent finding that long‐lived flies display higher levels of methionine suggests that regulation of lifespan depends on flux in the methionine cycle and levels of SAH/SAM, rather than absolute methionine levels. Although it is clear that each of the three branches of methionine metabolism—the methionine cycle, the transsulfuration pathway, and polyamine biosynthesis—plays a significant role in lifespan extension, how these branches crosstalk during regulation of lifespan is unknown. Moreover, how activity in these different branches of methionine metabolism change with age in different tissues and organs remains to be elucidated. ^13^C‐metabolic flux analysis (MFA) is a useful approach to determine cellular metabolic flux (Jin et al., 2004; Sauer, 2006). With this method, cells/organisms are fed isotope‐labeled nutrients, the labeling patterns of intracellular metabolites are measured, and computational methods are used to estimate flux (Zamboni, 2011). ^13^C‐metabolic flux methods to comprehensively quantify metabolic flux through the multiple methionine‐related pathways have been developed for mammalian cells (Shlomi, Fan, Tang, Kruger, & Rabinowitz, 2014). To achieve the strongest lifespan extension via manipulations of methionine metabolism, it will be important to quantify how metabolic flux through the multiple methionine‐related pathways changes with age in specific tissues and organs. Moreover, a full understanding of MetR is likely to require the identification of specific methyltransferases affected by changes in SAM and SAH. Recently, the set of CpG sites, the so‐called “epigenetic clocks,” has been used to predict chronological and biological age (Horvath, 2013); it will be interesting to ask if a specific signature of CpG sites predicts the MetR response. Furthermore, it will be of interest to explore whether noninvasive methods similar to Met‐PET can be used to evaluate the activity of methionine metabolism in humans and to predict the potential benefits of MetR for lifespan extension. Finally, given the high therapeutic potential, it will be interesting to search for drugs that can mimic the MetR response and extend lifespan via reprogramming of methionine metabolism.

## CONFLICT OF INTEREST

None declared.
